# Association of *APP* gene polymorphisms and promoter methylation with essential hypertension in Guizhou: a case–control study

**DOI:** 10.1186/s40246-023-00462-y

**Published:** 2023-03-20

**Authors:** Ruichao Li, Juhui Song, Ansu Zhao, Xiaoyan Diao, Ting Zhang, Xiaolan Qi, Zhizhong Guan, Yu An, Lingyan Ren, Chanjuan Wang, Yan He

**Affiliations:** 1 Key Laboratory of Endemic and Ethnic Diseases, Ministry of Education & Key Laboratory of Medical Molecular Biology of Guizhou Province, & Collaborative Innovation Center for Prevention and Control of Endemic and Ethnic Regional Diseases Co-constructed by the Province and Ministry, Guizhou Medical University, Guiyang, 550004 Guizhou China; 2 Department of Cardiovascular Medicine, Affiliated Hospital of Guizhou Medical University, Guiyang, China; 3 The Clinical Laboratory Center, Guizhou Provincial People’s Hospital, Guiyang, China; 4 Antenatal Diagnosis Centre, Guizhou Provincial People’s Hospital, Guiyang, China

**Keywords:** Essential hypertension, Amyloid precursor protein, Gene polymorphism, Promoter methylation, Guizhou minorities

## Abstract

**Background:**

Single-nucleotide polymorphisms (SNPs) and DNA methylation are crucial regulators of essential hypertension (EH). Amyloid precursor protein (*APP*) mutations are implicated in hypertension development. Nonetheless, studies on the association of *APP* gene polymorphism and promoter methylation with hypertension are limited. Therefore, this case–control aims to evaluate the genetic association of *APP* gene polymorphism and promoter methylation with EH in Guizhou populations.

**Objective and methods:**

We conducted a case–control study on 343 EH patients and 335 healthy controls (including Miao, Buyi, and Han populations) in the Guizhou province of China to analyze 11 single-nucleotide polymorphisms (rs2040273, rs63750921, rs2211772, rs2830077, rs467021, rs368196, rs466433, rs364048, rs364051, rs438031, rs463946) in the *APP* gene via MassARRAY SNP. The MassARRAY EpiTYPER was employed to detect the methylation levels of the promoters.

**Results:**

In the Han population, the rs2211772 genotype distribution was significantly different between disease and control groups (*χ*^2^ = 6.343, *P* = 0.039). The CC genotype reduced the risk of hypertension compared to the TT or TC genotype (OR 0.105, 95%CI 0.012–0.914, *P* = 0.041). For rs2040273 in the Miao population, AG or GG genotype reduced the hypertension risk compared with the AA genotype (OR 0.533, 95%CI 0.294–0.965, *P* = 0.038). Haplotype TCC (rs364051–rs438031–rs463946) increased the risk of EH in Guizhou (OR 1.427, 95%CI 1.020–1.996, *P* = 0.037). Each 1% increase in CpG_19 (− 613 bp) methylation level was associated with a 4.1% increase in hypertension risk (OR 1.041, 95%CI 1.002–1.081, *P* = 0.039). Each 1% increase in CpG_1 (− 296 bp) methylation level was associated with an 8% decrease in hypertension risk in women (OR 0.920, 95%CI 0.860–0.984, *P* = 0.015). CpG_19 significantly correlated with systolic blood pressure (*r* = 0.2, *P* = 0.03). The methylation levels of CpG_19 in hypertensive patients with rs466433, rs364048, and rs364051 minor alleles were lower than that with wild-type alleles (*P* < 0.05). Moreover, rs467021 and rs364051 showed strong synergistic interaction with EH (*χ*^2^ = 7.633, *P* = 0.006). CpG_11, CpG_19, and rs364051 showed weak synergistic interaction with EH (*χ*^2^ = 19.874, *P* < 0.001).

**Conclusion:**

In summary, rs2211772 polymorphism and promoter methylation level of *APP* gene may be linked to EH in Guizhou populations. Our findings will provide novel insights for genetic research of hypertension and Alzheimer's disease.

**Supplementary Information:**

The online version contains supplementary material available at 10.1186/s40246-023-00462-y.

## Introduction

In a national survey of hypertension in 2018, the prevalence of hypertension among Chinese adults was 24.7% (approximately 274 million). Hypertension is a serious health problem in China [1]. Notably, essential hypertension (EH) is a complex multifactorial disease. The effect of genetic factors on blood pressure changes varies from 30 to 50% and regulates blood pressure [2]. A single-nucleotide polymorphism (SNP) is a DNA sequence polymorphism caused by variation of a single nucleotide at a specific site in the genomic DNA sequence. In recent years, several SNPs associated with blood pressure have been reported [3]. DNA methylation also regulates blood pressure genes, potentially disrupting the phenotype and function of the vessel wall in response to environmental stress [4, 5]. Heritable epigenetic changes may partly explain the genetic absence of hypertension [6]. A genome-wide association study identified 12 novel loci of genetic variations associated with blood pressure. These sites showed differential DNA methylation in hypertension patients. DNA methylation may mediate the association of SNP variants with blood pressure [7].

The amyloid precursor protein (*APP*) is a gene located in the 21q21.3 region of human chromosomes and encodes the production of the amyloid precursor protein. β-Amyloid (Aβ) is a polypeptide produced via hydrolysis of amyloid precursor proteins mediated by β- and γ-secretases; it circulates in the blood, cerebrospinal fluid, and interstitial fluid [8]. *APP* gene mutations increase Aβ production and aggregation [9]. Aβ accumulation causes abnormal cerebral vascular metabolism, increased angiotensin II, and sympathetic nerve activity, resulting in hypertension [10]. The *APP* gene promoter region has a high frequency of CpG dinucleotides, increasing the possibility that DNA cytosine methylation participates in the regulation of *APP* expression [11]. Studies have shown that the CpG site on the *APP* gene promoter is hypomethylated in Alzheimer's disease (AD) of the brain, causing the overexpression of amyloid precursor protein, hence overproduction and abnormal metabolism of Aβ [12]. Significant methylation differences occur at 11 CpG sites in the *APP* gene between patients with traumatic brain injury and idiopathic normal pressure hydrocephalus [13]. Lead (Pb) potentially exerts neurotoxic effects by changing the overall methylation and promoter methylation patterns of the *APP* gene [14].

Nonetheless, reports on the association of *APP* gene polymorphism and promoter methylation with hypertension are limited. Therefore, we used the MassARRAY SNP (Agena Bioscience, Inc.) to detect 11 single-nucleotide polymorphisms (rs2040273, rs63750921, rs2211772, rs2830077, rs467021, rs368196, rs466433, rs364048, rs364051, rs438031, rs463946) in the *APP* gene under a case–control method. Notably, MassARRAY EpiTYPER detects methylation levels of promoters (GRCh38/hg38; chr21:26,171,035–26,171,512). This study aims to explore the genetic association of *APP* gene polymorphism and promoter methylation with EH in the Guizhou population and provide a theoretical basis for genetic pathogenesis, prevention as well as control approaches to EH.

## Results

### Basic demographic characteristics of the population

In different populations of Guizhou, we found no significant difference in gender and BMI between the hypertension group and the control group (*P* > 0.05). The average age of the hypertension group was greater than that of the control group, and this difference was statistically significant (*P* < 0.05) (Tables 1, 2).

**Table 1:** Basic characteristics of SNP research objects

Nations	Group	Gender (male/female)	Age (years)	Normal BMI [*n* (%)]^a^	Low BMI [*n* (%)]^b^	Overweight [*n* (%)]^b^	Obesity [*n* (%)]^b^
Total populations	Control (*n* = 335)	151/184	51.93 ± 13.91	176 (52.5)	17 (5.1)	105 (31.3)	37 (11.0)
	EH (*n* = 343)	164/179	58.87 ± 12.85	156 (45.5)	14 (4.1)	123 (35.9)	50 (14.6)
	*t*/*χ*^2^	0.511	− 6.744		0.038	2.619	3.031
	*P*	0.475	**< 0.001**		0.845	0.106	0.082
Miao population	Control (*n* = 111)	44/67	51.15 ± 15.92	55 (49.5)	5 (4.5)	39 (35.1)	12 (10.8)
	EH (*n* = 110)	58/52	57.91 ± 13.69	48 (43.6)	3 (2.7)	42 (38.2)	17 (15.5)
	*t*/*χ*^2^	3.808	− 3.381		0.248	0.500	1.308
	*P*	0.051	**0.001**		0.724	0.479	0.253
Buyi population	Control (*n* = 117)	52/65	51.63 ± 13.99	65 (55.6)	8 (6.8)	33 (28.2)	11 (9.4)
	EH (*n* = 119)	61/58	60.93 ± 12.91	58 (48.7)	8 (6.7)	41 (34.5)	12 (10.1)
	*t*/*χ*^2^	1.098	− 5.308		0.046	1.258	0.196
	*P*	0.295	**< 0.001**		0.830	0.262	0.658
Han population	Control (*n* = 107)	55/52	53.07 ± 11.40	56 (52.3)	4 (3.7)	33 (30.8)	14 (13.1)
	EH (*n* = 114)	45/69	57.63 ± 11.74	50 (43.9)	3 (2.6)	40 (35.1)	21 (18.4)
	*t*/*χ*^2^	3.17	− 2.925		0.049	1.005	1.733
	*P*	0.075	**0.004**		1.000	0.316	0.188

Bold indicates *P* < 0.05

Measurement data are expressed as mean ± SD, and Student’s t test is used for comparison between groups. Counting data are expressed as frequency (rate), and Chi-square test is used for comparison between groups

^a^BMI = kg/m^2^, Low BMI: BMI < 18.5, Normal BMI: 18.5 ≤ BMI < 24.0, Overweight: 24.0 ≤ BMI < 28.0, Obesity: BMI ≥ 28.0

^b^Compared with the normal BMI group

**Table 2:** Basic characteristics of methylation research subjects

	EH (*n* = 60)	Control (*n* = 59)	t/*χ*^2^	*P*
Gender (male/female)	30/30	30/29	0.009	0.926
Age (years)	59.12 ± 5.85	54.41 ± 6.13	− 4.287	**< 0.001**
Normal BMI [*n* (%)]	26 (43.3)	26 (44.1)		
Low BMI [*n* (%)]	2 (3.3)	3 (5.1)	0.000	1.000^a^
Overweight [*n* (%)]	27 (45.0)	22 (37.3)	0.263	0.608^a^
Obesity [*n* (%)]	5 (8.3)	8 (13.6)	0.555	0.456^a^

Bold indicates *P* < 0.05

Measurement data are expressed as mean ± SD, and Student’s t test is used for comparison between groups. Counting data are expressed as frequency (rate), and Chi-square test is used for comparison between groups

^a^Compared with the normal BMI group

### Risk factors for hypertension

A binary multivariate logistic regression model was constructed considering hypertension as the dependent variable and age, gender, as well as BMI as independent variables. For the general population of Guizhou, age, overweight, and obesity were independent risk factors for EH (OR 1.044, 95%CI 1.031–1.057, *P* = 0.001; OR 1.611, 95%CI 1.127–2.302, *P* = 0.009 and OR 2.065, 95%CI 1.248–3.418, *P* = 0.005) (Table 3).

**Table 3:** Risk factors of essential hypertension in Guizhou populations (*n* = 678)

Variables	B	SE	Wald	Adjusted OR (95% CI)^a^	*P*
Age (years)	0.043	0.006	47.049	1.044 (1.031–1.057)	**< 0.001**
Female				1	
Male	0.04	0.161	0.063	1.041 (0.759–1.429)	0.802
Normal BMI				1	
Low BMI	− 0.188	0.397	0.224	0.829 (0.381–1.804)	0.636
Overweight	0.477	0.182	6.859	1.611 (1.127–2.302)	**0.009**
Obesity	0.725	0.257	7.959	2.065 (1.248–3.418)	**0.005**

Bold indicates *P* < 0.05

*OR* odds ratio, *95%CI* 95% confidence interval

^a^Adjusted for age, sex, BMI

### Allele and genotype distribution

No mutant bases were detected at the rs63750921 site. The distributions of other SNPs were in accordance with the Hardy–Weinberg equilibrium (*P* > 0.05). We compared the allele and genotype frequencies of different SNPs of *APP* in different populations. The results revealed that the distribution of rs2211772 genotype between the disease group and the control group was significantly different in the Han population (*χ*^2^ = 6.343, *P* = 0.039). No significant difference was observed in the Miao and Buyi populations (*P* > 0.05). The distribution difference of rs2040273 allele and genotype between the disease group and control group was at a critical value in the Miao population (*χ*^2^ = 3.795, *P* = 0.051 and *χ*^2^ = 4.711, *P* = 0.095) (Additional file 1: Table S1).

### Genetic pattern analysis

Further, binary logistic regression analysis was used to analyze the inheritance pattern of rs2211772 associated with hypertension in the Han population and rs2040273 in the Miao population. After adjusting for age, gender, and BMI confounding factors, the CC genotype reduced the risk of hypertension for the rs2211772 in the Han population, compared with the TT or TC genotype (OR 0.105, 95%CI 0.012 -0.914, *P* = 0.041). AG or GG genotype reduced the hypertension risk for the rs2040273 in the Miao population, compared with the AA genotype (OR 0.533, 95%CI 0.294–0.965, *P* = 0.038) (Table 4).

**Table 4:** Genetic patterns analysis of *APP* in Guizhou Han population

SNP	Nation	Genetic patterns	Genotype	Control group	EH group	OR (95% CI)	*P*	Adjusted OR (95% CI)^a^	*P*
rs2211772	Han population	Codominat	TT	53 (50%)	64 (56%)	1		1	
			TC	46 (43%)	49 (43%)	0.882 (0.513–1.518)	0.651	0.955 (0.536–1.700)	0.875
			CC	8 (7%)	1 (1%)	0.104 (0.013–0.854)	**0.035**	0.103 (0.012–0.908)	**0.041**
		Dominant	TT	53 (50%)	64 (56%)	1		1	
			TC-CC	54 (50%)	50 (44%)	0.767 (0.452–1.302)	0.326	0.815 (0.466–1.428)	0.476
		Recessive	TT-TC	99 (93%)	113 (99%)	1		1	
			CC	8 (7%)	1 (1%)	0.11 (0.013–0.891)	**0.039**	0.105 (0.012–0.914)	**0.041**
		Overdominant	TT-CC	61 (57%)	65 (57%)	1		1	
			TC	46 (43%)	49 (43%)	1.00 (0.587–1.703)	0.999	1.07 (0.606–1.887)	0.816
		Log-additive	–	–	–	0.669 (0.42–1.065)	0.09	0.701 (0.43–1.143)	0.154
rs2040273	Miao population	Codominat	AA	31 (28%)	46 (42%)	1		1	
			AG	58 (52%)	47 (43%)	0.546 (0.301–0.991)	**0.047**	0.527 (0.28–0.991)	**0.047**
			GG	22 (20%)	17 (15%)	0.521 (0.239–1.136)	0.101	0.549 (0.24–1.259)	0.157
		Dominant	AA	31 (28%)	46 (42%)	1		1	
			AG-GG	80 (72%)	64 (58%)	0.539 (0.307–0.945)	**0.031**	0.533 (0.294–0.965)	**0.038**
		Recessive	AA-AG	89 (80%)	93 (85%)	1		1	
			GG	22 (20%)	17 (15%)	0.739 (0.369–1.484)	0.396	0.791 (0.376–1.664)	0.537
		Overdominant	AA-GG	53 (48%)	63 (57%)	1		1	
			AG	58 (52%)	47 (43%)	0.682 (0.401–1.159)	0.157	0.642 (0.364–1.131)	0.125
		Log-additive	–	–	–	0.689 (0.47–1.008)	0.055	0.699 (0.466–1.046)	0.082

Bold indicates *P* < 0.05

*OR* odds ratio, *95%CI* 95% confidence interval

^a^Adjust age, sex, BMI

### Linkage disequilibrium and haplotype analysis

**Fig. 1:**
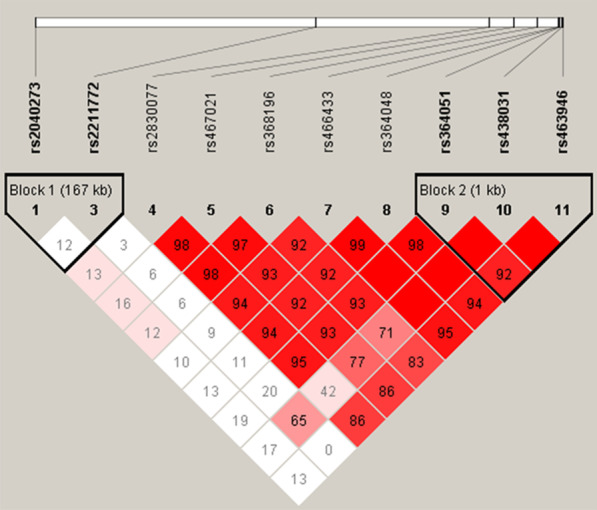
*APP* gene linkage disequilibrium analysis (*D*′). *D*′ = 0 meant complete linkage equilibrium, *D*′ = 1 meant complete linkage disequilibrium, and  > 0.8 meant strong linkage disequilibrium

Linkage disequilibrium analysis was performed on all SNPs using the Haploview 4.2 software (except for the rs63750921 where no mutation was detected). Figure 1 shows the results of linkage disequilibrium analysis for the general population of Guizhou. *D*′ = 0 indicated complete linkage equilibrium; *D*′ = 1 indicated complete linkage disequilibrium, and *D*′ > 0.8 implied strong linkage disequilibrium, which was represented by the red square. As shown, rs364051–rs438031–rs463946 had strong a linkage disequilibrium; rs2040273–rs2211772 had weak linkage disequilibrium. Haplotype analysis was performed using the SHEsis online software. Consequently, the haplotype TCC constructed with rs364051–rs438031–rs463946 increased the risk of EH in the Guizhou population (OR 1.427, 95%CI 1.020–1.996, *P* = 0.037); the one constructed with rs2040273–rs2211772 haplotype AC increased the risk of EH in Guizhou Miao population (OR 2.330, 95%CI 1.284–4.226, *P* = 0.004); GC increased the risk of EH in Guizhou Buyi population (OR 1.997, 95%CI 1.008–3.956, *P* = 0.044), (Table 5).

**Table 5:** Haplotype association analysis between EH group and control group in Guizhou population

Nation	Haplotype	EH group [*n* (%)] ^c^	Control group [*n* (%)]	*Χ*^2^	OR (95% CI)	*P*^d^
Total populations	AC^a^	94 (13.7)	93 (13.8)	0.005	0.989 (0.726–1.347)	0.943
	GC^a^	66 (9.6)	72 (10.8)	0.506	0.880 (0.619–1.251)	0.477
	TCC^b^	93 (13.5)	66 (9.9)	4.348	1.427 (1.020–1.996)	**0.037**
Miao population	AC^a^	38 (17.1)	18 (8.1)	8.071	2.330 (1.284–4.226)	**0.004**
	GC^a^	21 (9.7)	35 (15.7)	3.608	0.576 (0.324–1.023)	0.057
	TCC^b^	32 (14.5)	25 (11.1)	1.300	1.386 (0.789–2.435)	0.254
Buyi population	AC^a^	24 (10.1)	36 (15.6)	3.185	0.608 (0.351–1.054)	0.074
	GC^a^	26 (10.9)	13 (5.8)	4.053	1.997 (1.008–3.956)	**0.044**
	TCC^b^	24 (10.0)	16 (6.7)	1.594	1.530 (0.788–2.970)	0.207
Han population	AC^a^	32 (13.9)	38 (17.9)	1.352	0.738 (0.442–1.233)	0.245
	GC^a^	19 (8.5)	24 (11.1)	0.826	0.747 (0.397–1.404)	0.363
	TCC^b^	37 (16.2)	25 (11.9)	1.368	1.383 (0.802–2.385)	0.242

Bold indicates *P* < 0.05

^a^Haplotype SNP combination rs2040273–rs2211772

^b^Haplotype SNP combination rs364051–rs438031–rs463946

^c^All those frequency < 0.03 will be ignored in analysis

^d^The *P* value of Fisher exact tests

### The methylation level of *APP* gene promoter

**Fig. 2:**
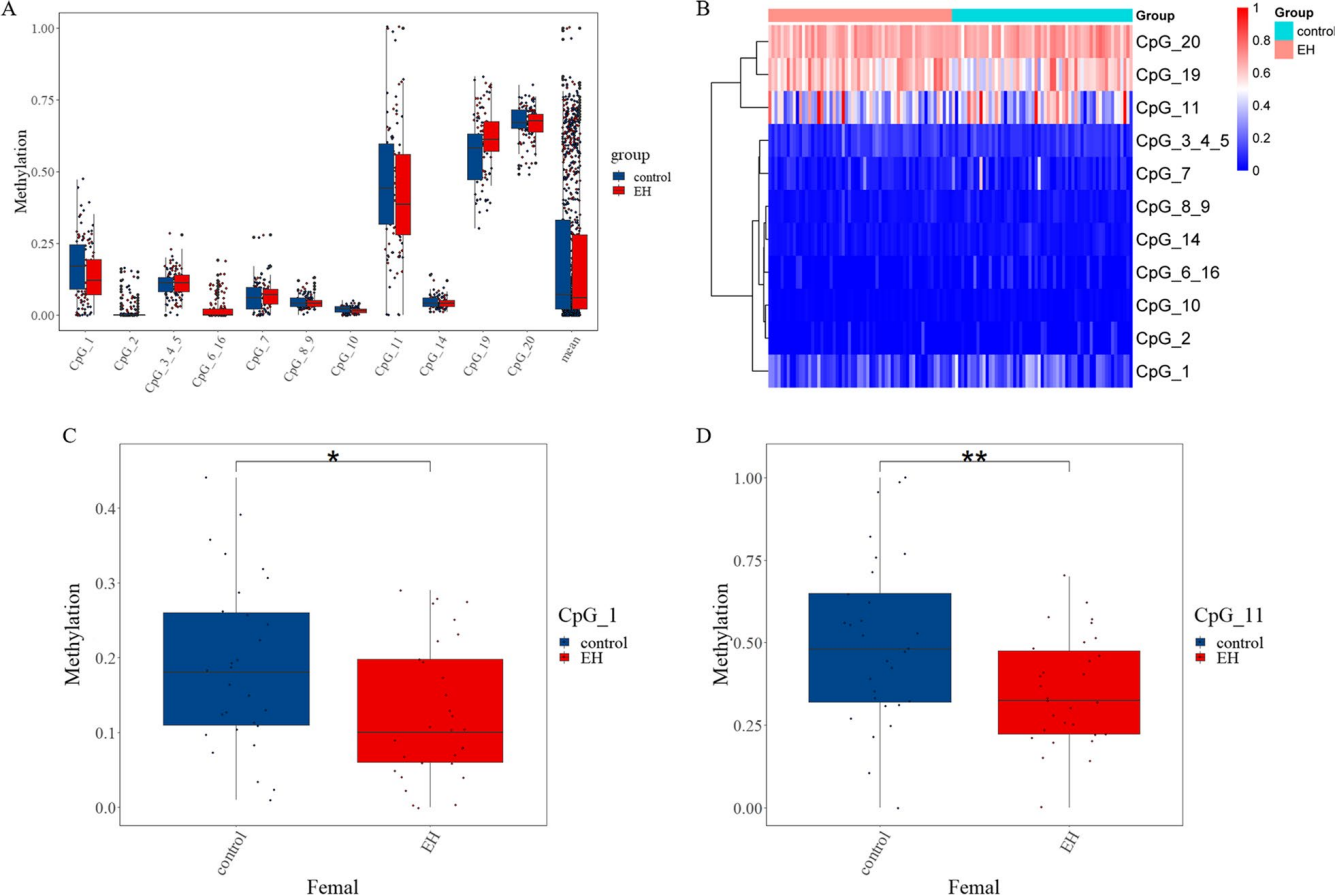
Analysis of methylation results. **A** Boxplot of methylation levels at CpG sites in the *APP* gene promoter (*n* = 119). **B** Heatmap of cluster analysis of methylation results (*n* = 119). **C** Differences in CpG_1 methylation level between women with hypertension and control groups (*n* = 59). **D** Differences in CpG_11 methylation level between women with hypertension and control groups (*n* = 59). *P < 0.05. **P < 0.01

The distribution of methylation results was skewed, hence expressed as medians (quartiles). The comparisons between groups were performed using the Mann–Whitney test. As a consequence, the methylation level of CpG_10 in the hypertension group was lower than that in the control group, whereas the methylation level of CpG_19 was higher than that in the control group (Fig. 2A). We found significant differences in the methylation levels of CpG_10 and CpG_19 between the hypertension group and the control group (*z* = − 2.024, *P* = 0.043 and *z* = − 2.721, *P* = 0.007) (Table 6). CpG_1 and CpG_11 were significantly different in the female hypertension group and control group (*P* = 0.018 and *P* = 0.009). The methylation levels in the hypertension group were lower than that in the control group (Fig. 2C/D). The heat map of cluster analysis revealed that CpG_11, CpG_19, and CpG_20 formed a cluster branch, all of which had high methylation levels (Fig. 2B).

**Table 6:** Methylation levels of CpG sites in *APP* gene promoter region

CpG sites	Position (negative chain)	Methylation level [Median, IQR (P25, P75)]		*z*	*P*
		Control group (*n* = 59)	EH group (*n* = 60)		
CpG_1	− 296 bp	0.17, 0.17 (0.08, 0.25)	0.12, 0.13 (0.07, 0.20)	− 1.662	0.096
CpG_2	− 316 bp	0.00, 0.00 (0.00, 0.00)	0.00, 0.00 (0.00, 0.00)	− 0.022	0.983
CpG_3_4_5	− 322 bp/− 332 bp/− 338 bp	0.11, 0.05 (0.08, 0.13)	0.11, 0.06 (0.08, 0.14)	− 0.837	0.402
CpG_6_16	− 355 bp/− 551 bp	0.00, 0.07 (0.00, 0.07)	0.00, 0.01 (0.00, 0.01)	− 1.603	0.109
CpG_7	− 361 bp	0.06, 0.08 (0.02, 0.10)	0.07, 0.06 (0.03, 0.09)	− 0.016	0.987
CpG_8_9	− 372 bp/− 381 bp	0.04, 0.03 (0.03, 0.06)	0.04, 0.02 (0.03, 0.05)	− 0.858	0.391
CpG_10	− 406 bp	0.02, 0.02 (0.01, 0.03)	0.01, 0.02 (0.00, 0.02)	− 2.024	**0.043**
CpG_11	− 416 bp	0.44, 0.29 (0.31, 0.60)	0.38, 0.28 (0.28, 0.56)	− 0.968	0.333
CpG_14	− 457 bp	0.04, 0.03 (0.03, 0.06)	0.04, 0.02 (0.03, 0.05)	− 1.04	0.298
CpG_19	− 613 bp	0.58, 0.16 (0.47, 0.63)	0.61, 0.11 (0.57, 0.68)	− 2.721	**0.007**
CpG_20	− 633 bp	0.67, 0.12 (0.65, 0.72)	0.67, 0.07 (0.63, 0.70)	− 1.068	0.285

Bold indicates *P* < 0.05

Quantitative skewed data are represented by Median (IQR), and comparisons between groups are performed using the Mann–Whitney test

### Association of *APP* gene promoter methylation with hypertension

**Fig. 3:**
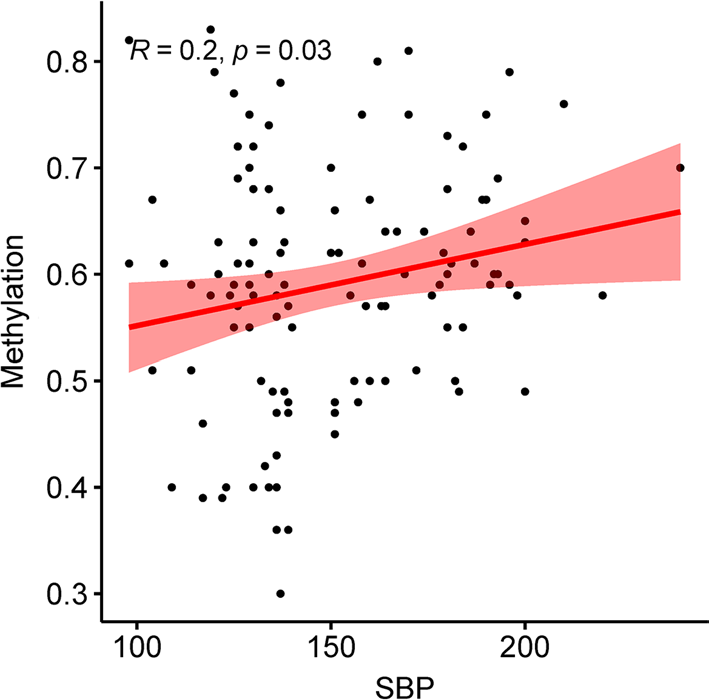
Correlation between *APP* gene CpG_19 and systolic blood pressure (*n* = 119)

Binary logistic regression analysis was performed on the methylation results. After adjusting for age, the results showed that for every 1% increase in CpG_10 methylation level, the risk of hypertension decreased by 32.4% (OR 0.676, 95%CI 0.467–0.977,* P* = 0.037). Every 1% increase in CpG_19 methylation level was associated with a 4.1% higher risk of hypertension (OR 1.041, 95%CI 1.002–1.081, *P* = 0.039). With every 1% increase in CpG_1 methylation level, the risk of hypertension in women was reduced by 8% (OR 0.920, 95%CI 0.860–0.984, *P* = 0.015) (Table 7). The correlation analysis between the methylation level of CpG sites and blood pressure showed a positive correlation between CpG_19 and systolic blood pressure (*r* = 0.2, *P* = 0.03) (Fig. 3).

**Table 7:** Binary logistic regression analysis of methylation results

CpG	B	SD	Wald	Adjusted OR (95% CI)^a^	*P*
CpG_1	− 0.040	0.02	3.905	0.961 (0.923–1.000)	**0.048**
CpG_2	− 0.010	0.109	0.009	0.990 (0.799–1.226)	0.924
CpG_3_4_5	0.043	0.043	1.033	1.044 (0.960–1.136)	0.309
CpG_6_16	− 0.118	0.059	3.959	0.889 (0.792–0.998)	**0.047**
CpG_7	0.008	0.039	0.047	1.008 (0.934–1.088)	0.829
CpG_8_9	− 0.063	0.087	0.513	0.939 (0.791–1.115)	0.474
CpG_10	− 0.392	0.188	4.333	0.676 (0.467–0.977)	**0.037**
CpG_11	− 0.003	0.009	0.147	0.997 (0.979–1.014)	0.701
CpG_14	− 0.076	0.1	0.577	0.927 (0.763–1.127)	0.447
CpG_19	0.040	0.019	4.269	1.041 (1.002–1.081)	**0.039**
CpG_20	− 0.054	0.037	2.127	0.948 (0.882–1.019)	0.145
CpG_1(female)	− 0.083	0.034	5.873	0.920 (0.860–0.984)	**0.015**
CpG_11(female)	− 0.025	0.015	2.746	0.975(0.946–1.005)	0.098

Bold indicates *P* < 0.05

^a^Adjust age

### Association of *APP* gene SNPs with methylation

**Fig. 4:**
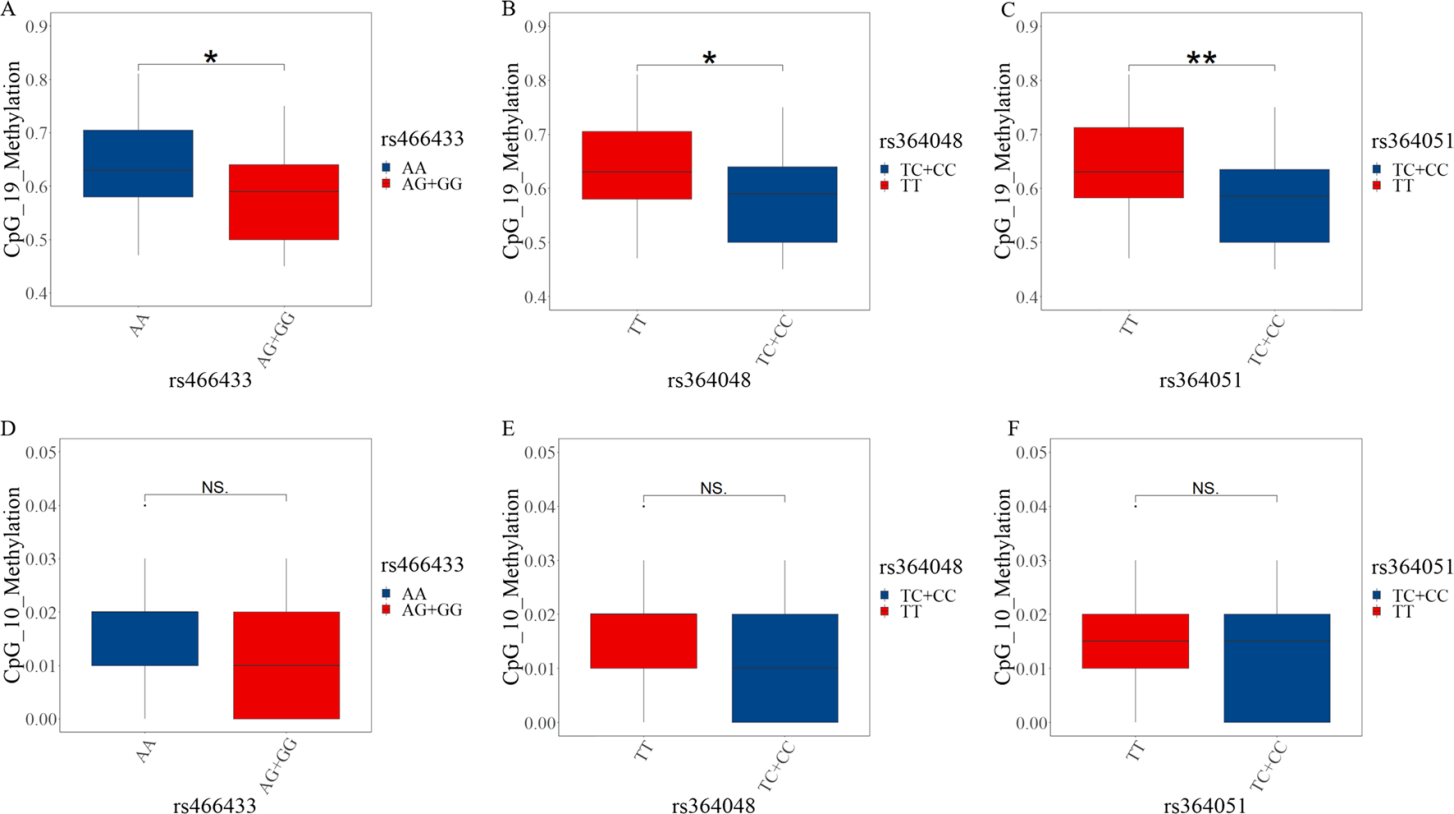
The relationship between *APP* gene promoter SNP genotype and methylation level in hypertensive patients (*n* = 60). *P < 0.05. **P < 0.01

The *APP* genes rs466433, rs364048, and rs364051 were located in the promoter region. We compared the differences in methylation levels of *APP* gene CpG_10 and CpG_19 in hypertensive patients with different genotypes. The methylation levels of CpG_19 in hypertensive patients carrying minor alleles of rs466433, rs364048, and rs364051 were lower than that of patients carrying wild-type alleles; the difference was statistically significant (Fig. 4).

### Interaction analysis of *APP* gene SNP and promoter methylation

**Fig. 5:**
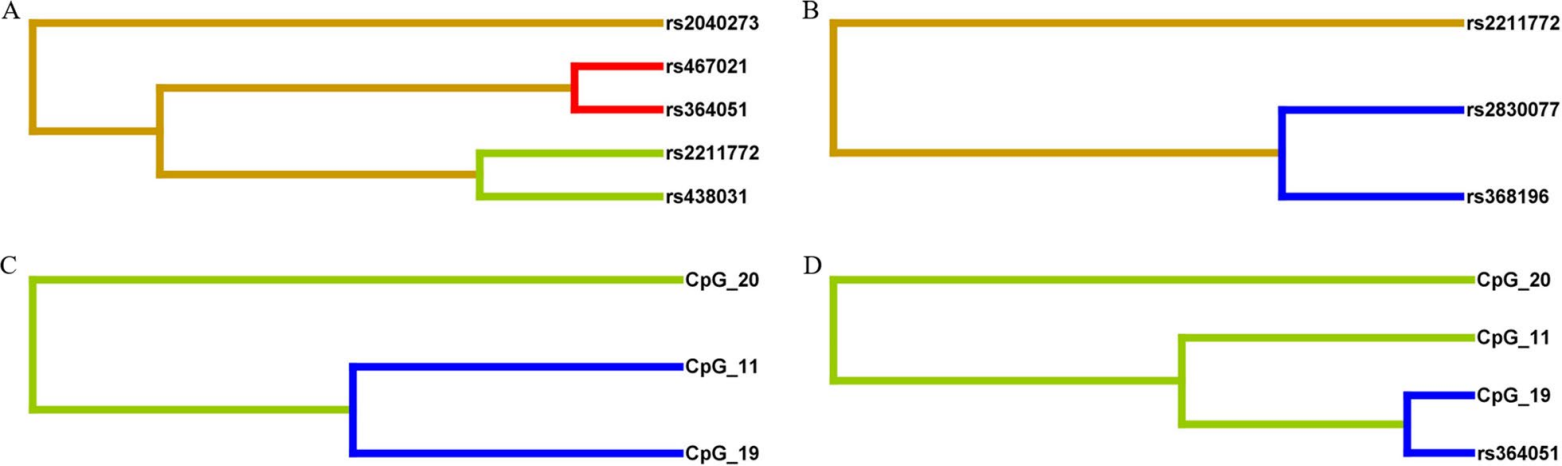
Different types of interaction dendrogram for SNPs and CpG sites.** A** SNP-SNP interaction in the general populations of Guizhou (*n* = 678). **B** SNP-SNP interaction in the Han population (*n* = 221). **C** CpG-CpG interaction in the Guizhou populations (*n* = 119). **D** SNP-CpG interaction in the Guizhou populations (*n* = 119). The dendrogram placed factors with strong interactions on the leaves. The color of the branches indicates the interaction from strong to weak (red, orange, green and blue). Red represents the highest degree of interaction or synergy, and blue represents low interaction or redundancy

Using the MDR3.0.2 software, we analyzed the interaction effects of SNP-SNP, CpG-CpG, and SNP-CpG, and established the optimal interaction model including 1–3 sites. For the general population of Guizhou, the best combination of two SNPs interaction was rs467021–rs364051, the cross-validation consistency was 7/10, and both the training balance accuracy and test balance accuracy were above 50%, and the model was statistically significant (*χ*^2^ = 7.633, *P* = 0.006). The interaction dendrogram of rs467021 and rs364051 was approached by red branches, indicating that rs467021 and rs364051 had a strong synergistic interaction on EH in Guizhou populations. Similarly, CpG_11, CpG_19 and CpG_20, CpG_11, CpG_19 and rs364051 interacted with EH in Guizhou population (*χ*^2^ = 15.575, *P* < 0.001 and *χ*^2^ = 19.874, *P* < 0.001) (Table 8 and Fig. 5).

**Table 8:** MDR analysis of SNPs and CpGs

Nation	Locus No	Best model	Training Bal. Acc	Testing Bal. Acc	Cross-validation Consistency	*χ*^2^	*P*^a^
	SNP-SNP interaction						
Total populations	1	rs438031	0.531	0.507	8/10	3.384	0.066
	2	rs467021, rs364051	0.554	0.522	7/10	7.633	**0.006**
	3	rs2040273, rs2211772, rs438031	0.579	0.513	4/10	15.877	**< 0.001**
Miao population	1	rs2040273	0.570	0.570	10/10	4.695	**0.030**
	2	rs2040273, rs2830077	0.588	0.480	5/10	5.602	**0.018**
	3	rs2040273, rs2211772, rs463946	0.625	0.497	8/10	12.995	**< 0.001**
Buyi population	1	rs467021	0.557	0.479	7/10	2.432	0.119
	2	rs467021, rs463946	0.593	0.484	3/10	7.765	**0.005**
	3	rs2040273, rs467021, rs364048	0.626	0.471	3/10	14.706	**< 0.001**
Han population	1	rs2211772	0.533	0.533	10/10	6.153	**0.013**
	2	rs2211772, rs368196	0.560	0.525	8/10	9.156	**0.003**
	3	rs2211772, rs2830077, rs368196	0.572	0.499	2/10	9.156	**0.003**
	CpG-CpG interaction						
Total populations	1	CpG_19	0.611	0.611	10/10	8.837	**0.003**
	2	CpG_19, CpG_20	0.654	0.586	8/10	13.769	**< 0.001**
	3	CpG_11, CpG_19, CpG_20	0.672	0.571	10/10	15.575	**< 0.001**
	CpG-SNP interaction						
Total populations	1	CpG_19	0.611	0.611	10/10	8.837	**0.003**
	2	CpG_19, CpG_20	0.658	0.545	5/10	13.769	**< 0.001**
	3	CpG_11, CpG_19, rs364051	0.703	0.545	8/10	19.874	**< 0.001**

Bold indicates *P* < 0.05

Training Bal. Acc., Training Balanced accuracy; Testing Bal. Acc, Testing Balanced accuracy; The training/testing balance accuracy represented the accuracy of the training set and the test set, and was used to evaluate the prediction error of the interaction model. Cross-validation consistency represented ten-fold cross-validation, comparing the number of times the same factor combination was determined, *N*/10 meant that *N* out of 10 cross-validations were significant. ^a^*P* value of permutation test, *P* < 0.05 means that the model is statistically significant

## Discussion

Hypertension is a multifactorial disease associated with both the environment and heredity [15]. Guizhou province is located in southwest China, with a subtropical monsoon climate, more rainfall, humid climate, and high air humidity. It is a cosmopolitan province, with Miao and Buyi being the two ethnic minorities having the largest population. Its unique geographical environment promotes the dietary preferences for sour, smoked, spicy, oil, and wine. Thus, investigating the genetic susceptibility of EH in different ethnic groups in Guizhou by a case–control method is of great importance. The MassARRAY flight mass spectrometry method is a technology that detects gene molecular weight with high accuracy, sensitivity, and throughput [16].

The *APP* gene is located in the 21q21.3 region of human chromosomes, with a full length of 290,579 bp, containing 20 exons, and encoding the amyloid precursor protein. APP is a transmembrane protein continuously cleaved by β and γ secretases (amyloid pathway) to produce polypeptides including Aβ40 and Aβ42 [8, 17]. Specifically, Aβ42 is prone to misfolding and forming aggregates [18]. *APP* gene mutations cause the occurrence of the amyloid pathway and increase Aβ production and aggregation [9]. Aβ aggregation causes abnormal cerebrovascular metabolism, increases angiotensin II and cerebrovascular resistance, and subsequently induces cerebrovascular dysfunction, resulting in decreased cerebral blood flow. To maintain cerebral perfusion in the face of these metabolic abnormalities, cerebral perfusion pressure must be increased, resulting in systemic hypertension. Meanwhile, Aβ40 causes the production of reactive oxygen species and/or downregulation of nitric oxide synthase through NADPH oxidase, which mediates an increase in sympathetic nerve activity, thereby increasing the total peripheral resistance and hypertension occurrence [10]. Thus, our case–control study for the first time explored the relationship between *APP* gene SNP mutation and hypertension. Consequently, we found that rs2211772 is associated with EH in the Guizhou Han population. Studies indicate that rs2211772 is associated with cholesterol and high-density lipoprotein [19], and plasma cholesterol level is a risk factor for cardiovascular disease [20], corroborating our findings. rs2211772 (chr21: 26027126, T > C) is an intronic variant of CpG-SNP, which generates a CpG site. Introns increase transcript levels by influencing the transcription rate, nuclear export, transcript stability, and mRNA translation efficiency [21]. Studies have shown that intronic SNP variants promote mRNA transcription, resulting in epigenetic gene modification [22, 23]. By predicting the transcription factors bound by the sequence where the SNP is located, we found that the sequence has several transcription factor binding sites including *TBX19*. It has been shown that TBX19 is a transcription factor that regulates growth and development as well as blood pressure [24]. Therefore, the binding activity of rs2211772 genotypes should be investigated using chromatin immunoprecipitation (ChIP) assay and the function of rs2211772 polymorphism needs to be explored using the luciferase reporter assay.

Lynn M Bekris reported that rs2040273 minor allele carriers have significantly lower levels of CSF Aβ42 [25]. Similarly, our study noted that the distribution of alleles and genotypes of rs2040273 in the Guizhou Miao population had a small statistical *P* value (*P* = 0.051 and *P* = 0.095) between the disease group and the control group. Moreover, regression analysis showed that in contrast with allele A, hypertension risk in carriers of minor allele G decreased (OR 0.533, 95%CI 0.294–0.965, *P* = 0.038). Thus, the relationship between rs2040273 and hypertension in a larger population is worth studying.

Elsewhere, Craig Myrum performed a functional evaluation of rs2830077 and found that the SNP is located in the active region of chromatin, which may have transcriptional enhancer activity and is a binding site for transcription factor CP2. Luciferase analysis revealed that the expression of its allele C improves *APP* expression [26]. However, we did not identify the relationship between rs2830077 and hypertension. rs63750921 mutation changes the encoded amino acid, and the pathological examination of the patient displayed severe cerebral amyloid angiopathy. This suggests that rs63750921 has vascular tropism [27]. This study, which for the first time reports rs63750921 mutation in a Chinese population, did not identify gene mutation of rs63750921. This indicates that the SNP is significantly conservative among the Chinese population.

Polygenic diseases including hypertension and diabetes often do not follow the common Mendelian inheritance pattern, where one gene modifies the phenotype of another gene, causing complex higher-order interactions between two or more genes [28]. Therefore, genetic interactions may induce hypertension risk. Our gene interaction analysis revealed that the interaction model made up of rs467021 and rs364051 had a cross-validation consistency of 7/10 (*P* = 0.006), and the interaction line was red. This indicates that rs467021 and rs364051 have a strong positive interaction effect on EH in the Guizhou populations, confirming the above standpoint.

Although human genome-wide association research has identified a large number of genetic loci associated with hypertension, these loci account for only a small fraction of its heritability [29]. Epigenetic modifications may partly explain the genetic absence of hypertension [6]. The *APP* promoter has multiple possible transcription factor binding sites [30, 31]. Promoter methylation has a strong correlation with transcriptional silencing of *APP* [32]. The *APP* proximal promoter region is crucial for cell-specific expression of the *APP* gene [33]. Herein, the methylation sequences (− 265 to − 742 bp) detected were predicted to contain 25 binding transcription factors, indicating that the target sequence promotes transcriptional regulation. Furthermore, we found that CpG_10 (− 406 bp), CpG_19 (− 613 bp), and CpG_1 (− 296 bp) of the target sequence were associated with hypertension. Regression analysis adjusted for confounding factors, and showed that for every 1% increase in CpG_10 methylation level, hypertension risk decreased by 32.4%; every 1% increase in CpG_19 methylation level was associated with a 4.1% higher risk of hypertension; every increase in CpG_1 methylation level 1%, the risk of hypertension in women reduced by 8%. This may be attributed to changes in methylation levels, which trigger changes in the sequence of transcription factor binding sites, hence affecting *APP* gene expression [34, 35] and abnormal metabolism of APP, ultimately resulting in hypertension [10].

Genetic variation potentially modulates DNA methylation [36]. SNP and CpG site methylation may jointly promote gene expression or alternative splicing, providing novel insights into polygenic disease research [37, 38]. This study included three SNPs in the positive regulatory region of the *APP* gene promoter, i.e., rs466433 (− 875 bp/T > C, generating a new CpG site), rs364048 (− 953 bp/A > C), rs364051 (− 1158/ A > C) [39]. Previous studies indicate that the transcriptional activity of haplotype TA (rs466433–rs364048) in neural cells is four times higher than that of haplotype CG, and *APP* mutation promoter upregulates *APP* gene expression and aggravates *Aβ* accumulation [40, 41]. Although we did not identify the relationship between promoter SNP variants and hypertension, the carriers of the minor allele of the promoter SNP in the hypertensive populations significantly reduced the CpG_19 methylation levels. Additionally, the results of MDR interaction analysis showed that CpG_11, CpG_19, and the promoter variant rs364051 interacted with EH in Guizhou populations. Thus, the variation of the *APP* gene promoter may influence gene expression by targeting the methylation level, hence changing the blood pressure.

Although hypertension and AD are closely related, the mechanism responsible for the association is not clear [42]. Animal experiments indicate that hypertension activates receptors for advanced glycation end products (RAGE) in the cerebrovascular system via oxidative stress, and mediates the transcytosis of Aβ across brain endothelial cells, resulting in Aβ accumulation, cognitive impairment, and memory degradation [43]. Previous research findings have also pointed out that hypertension promotes *APP* processing, which may be a mechanism of pathogenic interaction between hypertension and AD [44]. In this work, the polymorphism and methylation of the *APP* gene, closely related to AD, were associated with hypertension. This provides a genetic reference for further research on the interaction mechanism between hypertension and AD.

This work has the following limitations. First, due to sampling limitations, we did not identify cholesterol, triglyceride, and high-density lipoprotein, among other biochemical indicators contributing to hypertension for the Miao and Buyi populations, and therefore, these populations were not be included in the model for regression analysis. Secondly, environmental data, including smoking and diet, were lacking, and therefore, we could not analyze the interaction between genes and the environment. As such, the association of *APP* gene polymorphism and promoter methylation with EH deserves to be studied in a larger population with more comprehensive indicators.

In conclusion, we used Sequenom MassARRAY to investigate the associations of *APP* gene rs2040273, rs63750921, rs2211772, rs2830077, rs467021, rs368196, rs466433, rs364048, rs364051, rs438031, rs463946, and promoter methylation with EH in Guizhou populations. For the first time, we found that the *APP* gene rs2211772 and promoter methylation levels may be associated with EH among the Guizhou populations. Our findings provide an important reference value for a deeper understanding of genetic pathogenesis, prevention, and control strategies of EH in Guizhou.

## Materials and methods

### Subjects

This work adopted the group design method of simple random sampling. The outpatient, inpatient, and healthy physical examination population of the Affiliated Hospital of Guizhou Medical University and township health centers in Leishan and Libo counties were selected as the research objects between 2016 and 2018. In Guizhou's Miao and Buyi counties (Leishan County and Libo County), two townships and two villages in each township were randomly selected for a health quality survey. For patients with abnormal blood pressure in the survey, follow-up re-measurement was not less than 2 times. Eventually, 343 patients with EH were selected, including 110 Miao, 119 Buyi, and 114 Guiyang Han; and 335 healthy controls, including 111 Miao, 117 Buyi, and 107 Guiyang Han. At the same time, 60 patients with EH and 59 healthy controls were selected for methylation level detection. This study was approved by the Ethics Committee of the Affiliated Hospital of Guizhou Medical University, with the Approval Number: [2014] Lun Shen No. 45. All subjects voluntarily participated in the study and signed informed consent.

The inclusion criteria for the hypertension group were as follows: (1) meet the criteria of the 2010 Chinese Guidelines for the Prevention and Treatment of Hypertension: (2) age ≥ 18 years; blood pressure measured three times on different days, resting systolic blood pressure ≥ 140 mmHg (1 mm Hg = 0.133 kPa) and/or diastolic blood pressure ≥ 90 mmHg; (3) patients diagnosed with EH and currently taking antihypertensive drugs with normotensive blood pressure. All study subjects were Han, Miao, and Buyi people living in Guizhou, with no history of interracial marriage within three generations. Patients with secondary hypertension, congenital heart disease, cardiomyopathy, valvular disease, liver and kidney failure, pregnant women, substance abuse, or a history of mental illness were excluded.

The inclusion criteria for the control group are as follows. (1) systolic blood pressure < 140 mmHg and diastolic blood pressure < 90 mmHg; (2) no history of hypertension; (3) no antihypertensive drugs; all study subjects were Han, Miao, and Buyi people living in Guizhou, with no history of interracial marriage within three generations. Exclusion criteria were similar to that of the hypertension group.

### Basic information collection

All research subjects recorded basic information including nation, gender, age, blood pressure, height, and weight, and were calculated based on the “Guidelines for the Prevention and Control of Overweight and Obesity in Chinese Adults”; the body mass index (BMI) formula (BMI = kg/m^2^) divided the study subjects into low BMI (BMI < 18.5 kg/m^2^), normal BMI (18.5 kg/m^2^ ≤ BMI < 24.0 kg/m^2^), and overweight BMI (24.0 kg/m^2^ ≤ BMI < 28.0 kg/m^2^), BMI obesity (BMI ≥ 28.0 kg/m^2^).

### DNA extraction and quantification

A human peripheral blood DNA extraction kit (QIAGEN, Germany) was used to extract DNA from EDTA anticoagulated venous blood of the research subjects, and the concentration and purity were determined by NanoDrop2000 (Thermo Fisher Scientific Inc.). SNP detection requirements included: DNA concentration > 20 ng/μl, A_260_/A_280_ between 1.6 and 2.2; methylation detection requirements: DNA concentration > 20 ng/μl, A_260_/A_280_ between 1.6 and 2.2. DNA samples that did not meet the requirements were discarded.

### SNP determination and primer design and synthesis

In the dbSNP database [45], the SNP sites were identified in the exon, promoter, and 3-UTR regions, and function prediction website [46] was used to predict the function of SNPs. We also screened the Ensembl database [47] to select SNPs which minor allele frequencies (MAF) in the Han Chinese in southern China (CHS) that were greater than 5% as primary screening SNPs. In addition, the susceptible SNPs associated with increased Aβ aggregation or AD were annotated by consulting the PubMed database [48]. The UCSC database [49] was used to confirm the genomic homology of the gene sequence, and the location of SNPs, and to assess the potential risk of genotyping. Assay Designer 4.0 (Agena Bioscience, Inc) was used to evaluate the primer design of multiple SNPs, and the design parameters were adjusted based on different SNP information to meet the optimization criteria. Using the PAGE primer purification method, three primers corresponding to each SNP were synthesized, including two PCR primers and one single-base extension primer (Table 9).

**Table 9:** Primer sequences for *APP* gene SNP genotyping and methylation detection

SNP	Primer sequence
rs2040273	Amplify upstream primers: 5'-ACGTTGGATGGAGGAATGTAGTAGACCGAC-3'
	Amplify downstream primers: 5'-ACGTTGGATGCTCCATAACCAAACCAAACC-3'
	Single-base extension primer: 5'-CCAAACCCTTTCATATCATTT-3'
rs63750921	Amplify upstream primers: 5'-ACGTTGGATGGTGGGTTCAAACAAAGGTGC-3'
	Amplify downstream primers: 5'-ACGTTGGATGCAAGGTGATGACGATCACTG-3'
	Single-base extension primer: 5'-ggacCACCGCCCACCATGA-3'
rs2211772	Amplify upstream primers: 5'-ACGTTGGATGAGGGAGGAAATGACAGAGAG-3'
	Amplify downstream primers: 5'-ACGTTGGATGGTGCAAGTAGGTTGGATCTC-3'
	Single-base extension primer: 5'-aCTCATGATAGTCCATATTCAC-3'
rs2830077	Amplify upstream primers: 5'-ACGTTGGATGTGTCTCCTTATGGAGAGTGG-3'
	Amplify downstream primers: 5'-ACGTTGGATGAGTGTCTCTCTGAAGTGGTG-3'
	Single-base extension primer: 5'-cccctGTGACACGCTGACTTCCAGGCA-3'
rs467021	Amplify upstream primers: 5'-ACGTTGGATGGCATTGGTGCTTGTAATATC-3'
	Amplify downstream primers: 5'-ACGTTGGATGCTGCTTTCTTCTGACTTACC-3'
	Single-base extension primer: 5'-aACTTACCATGAGAATTCCA-3'
rs368196	Amplify upstream primers: 5'-ACGTTGGATGTTTCTTCCTCCACTGGACTG-3'
	Amplify downstream primers: 5'-ACGTTGGATGAGATCTAGAATCTGGGTGGG-3'
	Single-base extension primer: 5'-GGCTGTGAGTAAATAGAAAGGTA-3'
rs466433	Amplify upstream primers: 5'-ACGTTGGATGGATCATTCGTATTCGACCCC-3'
	Amplify downstream primers: 5'-ACGTTGGATGTCAGGACAGACACAATGAAG-3'
	Single-base extension primer: 5'-AACAAGGGCAGCGTT-3'
rs364048	Amplify upstream primers: 5'-ACGTTGGATGTTCTGCCATGCCACTTTCTC-3'
	Amplify downstream primers: 5'-ACGTTGGATGTGGGCAGTTCTAGAGCATTC-3'
	Single-base extension primer: 5'-gacttGACAGTGGACGGTTTGTGTTT-3'
rs364051	Amplify upstream primers: 5'-ACGTTGGATGTGCACTGCAGCCTGCCTTC-3'
	Amplify downstream primers: 5'-ACGTTGGATGAGGAAGGAAGTCTGTACCCC-3'
	Single-base extension primer: 5'-GTCAGCGCAATGAGCA-3'
rs438031	Amplify upstream primers: 5'-ACGTTGGATGCCCCATCCTAGTTTCAAGTG-3'
	Amplify downstream primers: 5'-ACGTTGGATGCCAGAAATGCCCAAAGATAG-3'
	Single-base extension primer: 5'-cagcAATGCCCAAAGATAGAATGCAC-3'
rs463946	Amplify upstream primers: 5'-ACGTTGGATGACTGTTGAAGGAAGTGCCTG-3'
	Amplify downstream primers: 5'-ACGTTGGATGCAAATTTGCCAGCGGTTTTC-3'
	Single-base extension primer: 5'-agcgtTTTTCATGCTACTTCTTCCT-3'
Methylation detection	Upstream primers: 5'-aggaagagagGGTTTGGTATTGTTTTTGTTGGT-3'
	Downstream primers: 5'-cagtaatacgactcactatagggagaaggctAAAAACTCCTAACTTCCTAAACTATCC-3'

### Determination of methylated region and primer design and synthesis

**Fig. 6:**
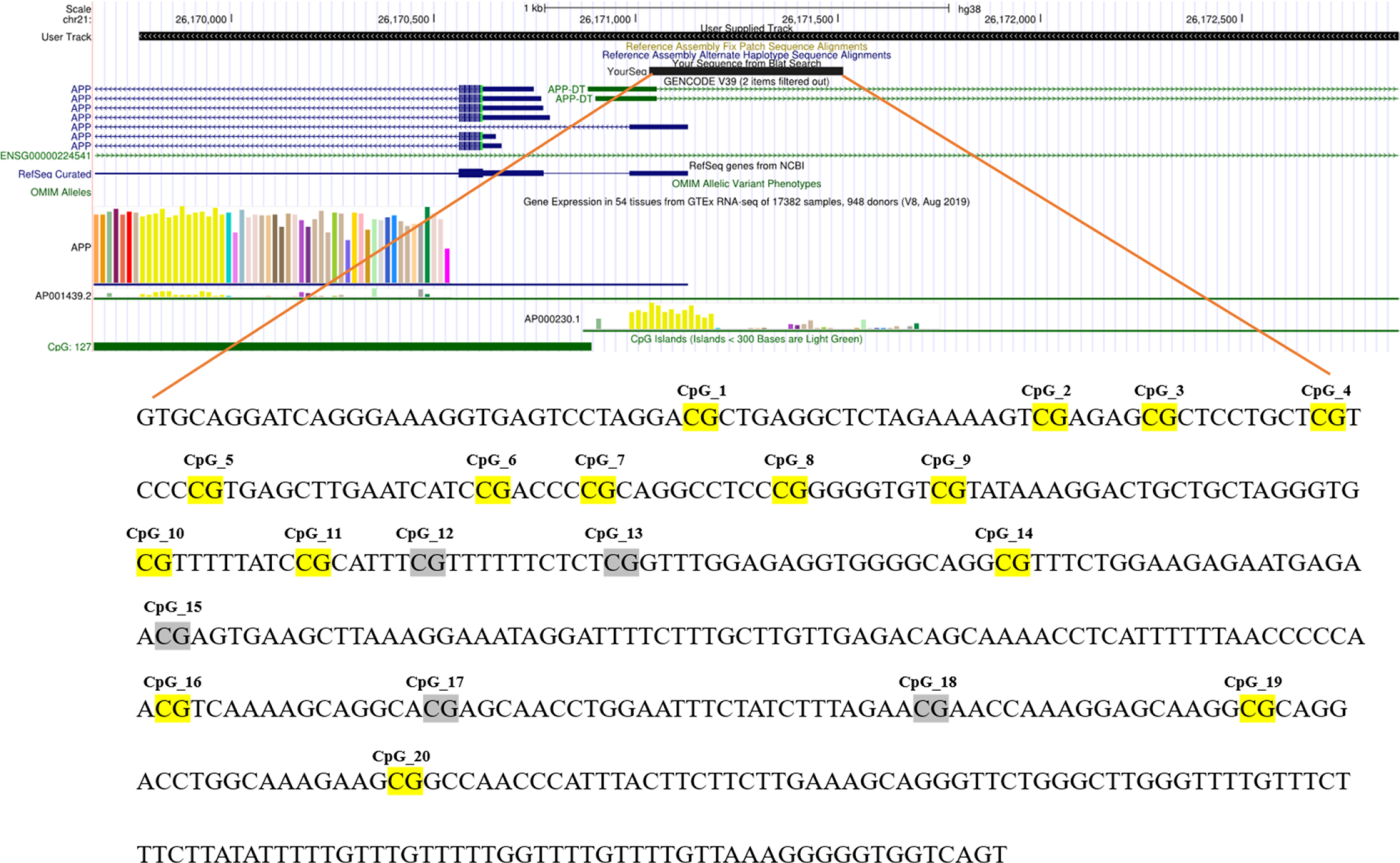
Schematic diagram of *APP* gene promoter CpG site. Location of CpG sites: chr21:26,171,035–26,171,512 (GRCh38/hg38). Detectable CpG sites were marked in yellow. Undetectable CpG sites were marked in gray. CpG_3, CpG_4 and CpG_5, CpG_6 and CpG_16, CpG_8 and CpG_9 were detected as a unit, respectively

The UCSC database was used to identify the promoter region of the *APP* gene, and the target sequence is located at chr21:26,171,035–26,171,512 (GRCh38/hg38), covering 478 bp, including 12 detectable CpG sites (Fig. 6). Transcription factors are proteins that bind DNA in a sequence-specific manner and regulate transcription, controlling chromatin and transcription by recognizing specific DNA sequences to form complex systems that direct genome expression. Potential transcription factors and binding sites of the target sequence were identified through the Promo database [50], including 25 predicted transcription factors associated with the expression of the *APP* gene, with positive or negative regulatory relationships. Primer protocol design for targeting sequences was performed using Agena EpiDesigner (Agena Bioscience, Inc.). PCR primer sequences of the corresponding fragments were synthesized using the PAGE primer purification method (Table 9).

### Genotyping and methylation testing

PCR amplification reaction, shrimp alkaline phosphatase reaction, single-base extension reaction (for SNP detection) or transcriptase cleavage reaction (for methylation detection), resin purification, chip spotting, and mass spectrometry detection were sequentially performed using the MassARRAY detection platform. The TYPER 4.0 (Agena Bioscience, Inc) was used to collect the original data, genotyping map, and other test results. The sequences of CpG sites between two bases A were digested to obtain small fragments with similar molecular weight, then these CpG sites were combined and detected, and the result was the average methylation degree of these CpG sites. Therefore, CpG_3, CpG_4, and CpG_5; CpG_6 and CpG_16; CpG_8 and CpG_9 were detected as a unit.

### Statistical analysis

Statistical analyses were performed using the SPSS 26.0 software (IBM Corp., Armonk, NY, USA). Normal measurement data were expressed as mean ± standard deviation (mean ± sd), and comparison between groups was performed by the Student's t test; skewed measurement data were represented by [Median, IQR (P25, P75)], and comparison between groups was performed by Mann–Whitney test. The count data were expressed as frequency (constituent ratio), and the comparison between groups was performed using the Chi-square test or Fisher's exact test. Binary logistic regression was used to analyze the relationship between SNPs and EH. Allele and genotype frequencies and the Hardy–Weinberg equilibrium test were calculated using the SNPStats online software [51]. Linkage disequilibrium analysis was performed using the Haploview 4.2 software. The SHEsis online software [52] was used to construct haplotypes and calculate the odds ratio (OR) and 95% confidence interval (CI). SNP-SNP, CpG-CpG, and CpG-SNP interaction analyses were performed using the MDR3.0.2 software. Methylation results were plotted using the R software (version 3.6.2, R Foundation for Statistical Computing, Vienna, Austria). A two-sided test *P* < 0.05 indicated a statistically significant difference.

## Supplementary Information

**Additional file 1: Table S1.** *APP* allele and genotype distribution.

## Abbreviations

EH: Essential hypertension
APP: Amyloid precursor protein
SNP: Single-nucleotide polymorphism
Aβ: β-Amyloid
AD: Alzheimer's disease
BMI: Body mass index
OR: Odds ratio
95%CI: 95% Confidence interval
MAF: Minor Allele frequencies
