# Supplementary material for: Association of APP gene polymorphisms and promoter methylation with essential hypertension in Guizhou: a case–control study
Source: Hum Genomics. 2023 Mar 20;17:25. doi: 10.1186/s40246-023-00462-y (PMC10026478; doi:10.1186/s40246-023-00462-y)
Supplement: Supplementary file 1 — Additional file 1: Table S1. APP allele and genotype distribution. [file 40246_2023_462_MOESM1_ESM.docx]

**Additional file 1: Table S1**

| **Table S1** *APP* allele and genotype distribution | | | | | | | | | | | | | | | | | |
| --- | --- | --- | --- | --- | --- | --- | --- | --- | --- | --- | --- | --- | --- | --- | --- | --- | --- |
| **SNPs** | **Allele/ Genotype** | **Total populations** | | ***P ^a^*** | ***P ^b^*** | **Miao population** | | ***P ^a^*** | ***P ^b^*** | **Buyi population** | | ***P ^a^*** | ***P ^b^*** | **Han population** | | ***P ^a^*** | *P ^b^* |
|  |  | **CO (n=335)** | **EH (n=343)** |  |  | **CO (n=111)** | **EH (n=110)** |  |  | **CO (n=117)** | **EH (n=119)** |  |  | **CO (n=107)** | **EH (n=114)** |  |  |
| rs2040273 | A | 442(66%) | 451(66%) | 0.954 | 0.964 | 120(54%) | 139(63%) | 0.051 | 0.095 | 171(73%) | 162(68%) | 0.233 | 0.345 | 151(71%) | 150(66%) | 0.282 | 0.344 |
|  | G | 228(34%) | 235(34%) |  |  | 102(46%) | 81(37%) |  |  | 63(27%) | 76(32%) |  |  | 63(29%) | 78(34%) |  |  |
|  | AA | 146(44%) | 150(44%) |  |  | 31(28%) | 46(42%) |  |  | 64(55%) | 54(45%) |  |  | 51(48%) | 50(44%) |  |  |
|  | AG | 150(45%) | 151(44%) |  |  | 58(52%) | 47(43%) |  |  | 43(37%) | 54(45%) |  |  | 49(46%) | 50(44%) |  |  |
|  | GG | 39(12%) | 42(12%) |  |  | 22(20%) | 17(15%) |  |  | 10(9%) | 11(9%) |  |  | 7(7%) | 14(12%) |  |  |
|  | *P_hwe_* | 1.000 | 0.720 |  |  | 0.700 | 0.410 |  |  | 0.480 | 0.830 |  |  | 0.360 | 0.840 |  |  |
| rs63750921 | G | 670(100%) | 686(100%) | NA | NA | 222(100%) | 220(100%) | NA | NA | 234(100%) | 238(100%) | NA | NA | 214(100%) | 228(100%) | NA | NA |
|  | C | 0(0%) | 0(0%) |  |  | 0(0%) | 0(0%) |  |  | 0(0%) | 0(0%) |  |  | 0(0%) | 0(0%) |  |  |
|  | GG | 335(100%) | 343(100%) |  |  | 111(100%) | 110(100%) |  |  | 117(100%) | 119(100%) |  |  | 107(100%) | 114(100%) |  |  |
|  | GC | 0(0%) | 0(0%) |  |  | 0(0%) | 0(0%) |  |  | 0(0%) | 0(0%) |  |  | 0(0%) | 0(0%) |  |  |
|  | CC | 0(0%) | 0(0%) |  |  | 0(0%) | 0(0%) |  |  | 0(0%) | 0(0%) |  |  | 0(0%) | 0(0%) |  |  |
|  | *P_hwe_* | NA | NA |  |  | NA | NA |  |  | NA | NA |  |  | NA | NA |  |  |
| rs2211772 | T | 505(75%) | 526(77%) | 0.574 | 0.069 | 169(76%) | 161(73%) | 0.477 | 0.205 | 184(79%) | 188(79%) | 0.924 | 0.747 | 152(71%) | 177(78%) | 0.112 | **0.039** |
|  | C | 165(25%) | 160(23%) |  |  | 53(24%) | 59(27%) |  |  | 50(21%) | 50(21%) |  |  | 62(29%) | 51(22%) |  |  |
|  | TT | 194(58%) | 195(57%) |  |  | 68(61%) | 58(53%) |  |  | 73(62%) | 73(61%) |  |  | 53(50%) | 64(56%) |  |  |
|  | TC | 117(35%) | 136(40%) |  |  | 33(30%) | 45(41%) |  |  | 38(32%) | 42(35%) |  |  | 46(43%) | 49(43%) |  |  |
|  | CC | 24(7%) | 12(3%) |  |  | 10(9%) | 7(6%) |  |  | 6(5%) | 4(3%) |  |  | 8(7%) | 1(1%) |  |  |
|  | *P_hwe_* | 0.300 | 0.050 |  |  | 0.066 | 0.810 |  |  | 0.780 | 0.590 |  |  | 0.810 | 0.013 |  |  |
| rs2830077 | C | 376(56%) | 388(57%) | 0.870 | 0.839 | 129(58%) | 130(59%) | 0.834 | 0.671 | 138(59%) | 138(58%) | 0.827 | 0.442 | 109(51%) | 120(53%) | 0.721 | 0.521 |
|  | A | 294(44%) | 298(43%) |  |  | 93(42%) | 90(41%) |  |  | 96(41%) | 100(42%) |  |  | 105(49%) | 108(47%) |  |  |
|  | CC | 110(33%) | 118(34%) |  |  | 39(35%) | 43(39%) |  |  | 38(32%) | 42(35%) |  |  | 33(31%) | 33(29%) |  |  |
|  | CA | 156(47%) | 152(44%) |  |  | 51(46%) | 44(40%) |  |  | 62(53%) | 54(45%) |  |  | 43(40%) | 54(47%) |  |  |
|  | AA | 69(21%) | 73(21%) |  |  | 21(19%) | 23(21%) |  |  | 17(15%) | 23(19%) |  |  | 31(29%) | 27(24%) |  |  |
|  | *P_hwe_* | 0.320 | 0.078 |  |  | 0.560 | 0.076 |  |  | 0.350 | 0.460 |  |  | 0.052 | 0.580 |  |  |
| rs467021 | C | 391(58%) | 401(58%) | 0.971 | 0.905 | 133(60%) | 132(60%) | 0.985 | 0.939 | 143(61%) | 146(61%) | 0.958 | 0.265 | 115(54%) | 123(54%) | 0.965 | 0.501 |
|  | A | 279(42%) | 285(42%) |  |  | 89(40%) | 88(40%) |  |  | 91(39%) | 92(39%) |  |  | 99(46%) | 105(46%) |  |  |
|  | CC | 116(35%) | 122(36%) |  |  | 42(38%) | 43(39%) |  |  | 40(34%) | 47(39%) |  |  | 34(32%) | 32(28%) |  |  |
|  | CA | 159(47%) | 157(46%) |  |  | 49(44%) | 46(42%) |  |  | 63(54%) | 52(44%) |  |  | 47(44%) | 59(52%) |  |  |
|  | AA | 60(18%) | 64(19%) |  |  | 20(18%) | 21(19%) |  |  | 14(12%) | 20(17%) |  |  | 26(24%) | 23(20%) |  |  |
|  | *P_hwe_* | 0.650 | 0.320 |  |  | 0.430 | 0.170 |  |  | 0.180 | 0.440 |  |  | 0.240 | 0.710 |  |  |
| rs368196 | C | 393(59%) | 407(59%) | 0.801 | 0.947 | 133(60%) | 135(61%) | 0.754 | 0.872 | 145(62%) | 145(61%) | 0.816 | 0.418 | 115(54%) | 127(56%) | 0.679 | 0.419 |
|  | T | 277(41%) | 279(41%) |  |  | 89(40%) | 85(39%) |  |  | 89(38%) | 93(39%) |  |  | 99(46%) | 101(44%) |  |  |
|  | CC | 122(36%) | 129(38%) |  |  | 43(39%) | 46(42%) |  |  | 43(37%) | 47(39%) |  |  | 36(34%) | 36(32%) |  |  |
|  | CT | 149(44%) | 149(43%) |  |  | 47(42%) | 43(39%) |  |  | 59(50%) | 51(43%) |  |  | 43(40%) | 55(48%) |  |  |
|  | TT | 64(19%) | 65(19%) |  |  | 21(19%) | 21(19%) |  |  | 15(13%) | 21(18%) |  |  | 28(26%) | 23(20%) |  |  |
|  | *P_hwe_* | 0.140 | 0.073 |  |  | 0.240 | 0.071 |  |  | 0.560 | 0.330 |  |  | 0.052 | 0.850 |  |  |
| rs466433 | A | 553(83%) | 568(83%) | 0.899 | 0.960 | 192(86%) | 186(85%) | 0.562 | 0.615 | 187(80%) | 194(82%) | 0.660 | 0.909 | 174(81%) | 188(82%) | 0.754 | 0.928 |
|  | G | 117(17%) | 118(17%) |  |  | 30(14%) | 34(15%) |  |  | 47(20%) | 44(18%) |  |  | 40(19%) | 40(18%) |  |  |
|  | AA | 228(68%) | 234(68%) |  |  | 84(76%) | 78(71%) |  |  | 74(63%) | 79(66%) |  |  | 70(65%) | 77(68%) |  |  |
|  | AG | 97(29%) | 100(29%) |  |  | 24(22%) | 30(27%) |  |  | 39(33%) | 36(30%) |  |  | 34(32%) | 34(30%) |  |  |
|  | GG | 10(3%) | 9(3%) |  |  | 3(3%) | 2(2%) |  |  | 4(3%) | 4(3%) |  |  | 3(3%) | 3(3%) |  |  |
|  | *P_hwe_* | 1.000 | 0.850 |  |  | 0.410 | 1.000 |  |  | 1.000 | 1.000 |  |  | 1.000 | 1.000 |  |  |
| rs364048 | T | 549(82%) | 565(82%) | 0.840 | 0.765 | 192(86%) | 186(85%) | 0.562 | 0.615 | 186(79%) | 195(82%) | 0.501 | 0.810 | 171(80%) | 184(81%) | 0.834 | 0.941 |
|  | C | 121(18%) | 121(18%) |  |  | 30(14%) | 34(15%) |  |  | 48(21%) | 43(18%) |  |  | 43(20%) | 44(19%) |  |  |
|  | TT | 227(68%) | 232(68%) |  |  | 84(76%) | 78(71%) |  |  | 74(63%) | 80(67%) |  |  | 69(64%) | 74(65%) |  |  |
|  | TC | 95(28%) | 101(29%) |  |  | 24(22%) | 30(27%) |  |  | 38(32%) | 35(29%) |  |  | 33(31%) | 36(32%) |  |  |
|  | CC | 13(4%) | 10(3%) |  |  | 3(3%) | 2(2%) |  |  | 5(4%) | 4(3%) |  |  | 5(5%) | 4(4%) |  |  |
|  | *P_hwe_* | 0.460 | 1.000 |  |  | 0.410 | 1.000 |  |  | 1.000 | 1.000 |  |  | 0.760 | 1.000 |  |  |
| rs364051 | T | 531(79%) | 547(80%) | 0.825 | 0.929 | 182(82%) | 176(80%) | 0.595 | 0.812 | 180(77%) | 191(80%) | 0.378 | 0.564 | 169(79%) | 180(79%) | 0.995 | 0.971 |
|  | C | 139(21%) | 139(20%) |  |  | 40(18%) | 44(20%) |  |  | 54(23%) | 47(20%) |  |  | 45(21%) | 48(21%) |  |  |
|  | TT | 210(63%) | 219(64%) |  |  | 76(68%) | 71(65%) |  |  | 68(58%) | 77(65%) |  |  | 66(62%) | 71(62%) |  |  |
|  | TC | 111(33%) | 109(32%) |  |  | 30(27%) | 34(31%) |  |  | 44(38%) | 37(31%) |  |  | 37(35%) | 38(33%) |  |  |
|  | CC | 14(4%) | 15(4%) |  |  | 5(5%) | 5(5%) |  |  | 5(4%) | 5(4%) |  |  | 4(4%) | 5(4%) |  |  |
|  | *P_hwe_* | 1.000 | 0.740 |  |  | 0.340 | 0.760 |  |  | 0.610 | 0.780 |  |  | 1.000 | 1.000 |  |  |
| rs438031 | T | 601(90%) | 593(86%) | 0.064 | 0.141 | 197(89%) | 188(85%) | 0.303 | 0.394 | 218(93%) | 214(90%) | 0.205 | 0.184 | 186(87%) | 191(84%) | 0.351 | 0.498 |
|  | C | 69(10%) | 93(14%) |  |  | 25(11%) | 32(15%) |  |  | 16(7%) | 24(10%) |  |  | 28(13%) | 37(16%) |  |  |
|  | TT | 268(80%) | 254(74%) |  |  | 86(77%) | 80(73%) |  |  | 101(86%) | 95(80%) |  |  | 81(76%) | 79(69%) |  |  |
|  | TC | 65(19%) | 85(25%) |  |  | 25(23%) | 28(25%) |  |  | 16(14%) | 24(20%) |  |  | 24(22%) | 33(29%) |  |  |
|  | CC | 2(1%) | 4(1%) |  |  | 0(0%) | 2(2%) |  |  | 0(0%) | 0(0%) |  |  | 2(2%) | 2(2%) |  |  |
|  | *P_hwe_* | 0.550 | 0.360 |  |  | 0.350 | 1.000 |  |  | 1.000 | 0.600 |  |  | 1.000 | 0.730 |  |  |
| rs463946 | C | 547(82%) | 562(82%) | 0.893 | 0.480 | 193(87%) | 183(83%) | 0.268 | 0.592 | 186(79%) | 196(82%) | 0.428 | 0.715 | 168(79%) | 183(80%) | 0.648 | 0.335 |
|  | G | 123(18%) | 124(18%) |  |  | 29(13%) | 37(17%) |  |  | 48(21%) | 42(18%) |  |  | 46(21%) | 45(20%) |  |  |
|  | CC | 228(68%) | 230(67%) |  |  | 85(77%) | 77(70%) |  |  | 75(64%) | 81(68%) |  |  | 68(64%) | 72(63%) |  |  |
|  | CG | 91(27%) | 102(30%) |  |  | 23(21%) | 29(26%) |  |  | 36(31%) | 34(29%) |  |  | 32(30%) | 39(34%) |  |  |
|  | GG | 16(5%) | 11(3%) |  |  | 3(3%) | 4(4%) |  |  | 6(5%) | 4(3%) |  |  | 7(7%) | 3(3%) |  |  |
|  | *P_hwe_* | 0.099 | 1.000 |  |  | 0.390 | 0.500 |  |  | 0.570 | 0.760 |  |  | 0.250 | 0.560 |  |  |
| Measurement data are compared using chi-square test or Fisher's exact test. EH: Hypertension group, CO: Control group. P_hwe_: Hardy-Weinberg equilibrium test. a: Allele *P* value; b: Genotype *P* value. | | | | | | | | | | | | | | | | | |
